# Biochemistry of microbial itaconic acid production

**DOI:** 10.3389/fmicb.2013.00023

**Published:** 2013-02-14

**Authors:** Matthias G. Steiger, Marzena L. Blumhoff, Diethard Mattanovich, Michael Sauer

**Affiliations:** ^1^Austrian Centre of Industrial Biotechnology (ACIB GmbH)Vienna, Austria; ^2^Department of Biotechnology, BOKU – Vienna Institute of BioTechnology, University of Natural Resources and Life SciencesVienna, Austria; ^3^School of Bioengineering, University of Applied Sciences FH-Campus WienVienna, Austria

**Keywords:** *cis*-aconitic acid decarboxylase, *Aspergillus terreus*, *Aspergillus niger*, metabolic engineering, biochemical pathways, microbial organic acid production, industrial microbiology

## Abstract

Itaconic acid is an unsaturated dicarbonic acid which has a high potential as a biochemical building block, because it can be used as a monomer for the production of a plethora of products including resins, plastics, paints, and synthetic fibers. Some *Aspergillus* species, like *A. itaconicus* and *A. terreus,* show the ability to synthesize this organic acid and *A. terreus* can secrete significant amounts to the media (>80 g/L). However, compared with the citric acid production process (titers >200 g/L) the achieved titers are still low and the overall process is expensive because purified substrates are required for optimal productivity. Itaconate is formed by the enzymatic activity of a *cis*-aconitate decarboxylase (CadA) encoded by the *cadA* gene in *A. terreus*. Cloning of the *cadA *gene into the citric acid producing fungus *A. niger* showed that it is possible to produce itaconic acid also in a different host organism. This review will describe the current status and recent advances in the understanding of the molecular processes leading to the biotechnological production of itaconic acid.

## INTRODUCTION

Itaconic acid (2-methylidenebutanedioic acid) is an unsaturated di-carbonic acid. It has a broad application spectrum in the industrial production of resins and is used as a building block for acrylic plastics, acrylate latexes, super-absorbents, and anti-scaling agents (Willke and Vorlop, 2001; Okabe et al., 2009). Since the 1960s the production of itaconic acid is achieved by the fermentation with *Aspergillus terreus* on sugar containing media (Willke and Vorlop, 2001). Although also other microorganisms like *Ustilago zeae *(Haskins et al., 1955), *U. maydis*, *Candida* sp. (Tabuchi et al., 1981), and *Rhodotorula* sp. (Kawamura et al., 1981) were found to produce itaconic acid, *A. terreus* is still the dominant production host, because so far only bred strains of this species can reach levels of up to 80–86 g/L (Okabe et al., 2009; Kuenz et al., 2012). Since the 1990s, itaconic acid as a renewable material is attracting a lot of interest. Although the production costs for itaconic acid are declining in the last years ($ 4 per kg in 2001; Willke and Vorlop, 2001), it is still a valuable product with an estimated price of $ 2 per kg. Currently, the worldwide production capacity of itaconic acid is expected to be about 50 kt per year, facing a demand of about 30 kt (Shaw, 2013, Itaconix Corporation, personal communication). Especially, for the production of polymers it is of interest, because in the future it can function as a substitute for acrylic and methacrylic acid used for the production of plastics (Okabe et al., 2009). However, these applications require an even lower price of the starting material. The current knowledge about the biotechnological production of itaconic acid was recently reviewed (Willke and Vorlop, 2001; Okabe et al., 2009). The latter review covers the industrial production of itaconic acid and the applications of this product. Therefore, we focus in this report on the recent advances with an emphasis on the biochemistry of the process and new genetic engineering targets. For rational strain improvement, it is essential to understand the underlying biological concepts and biochemical pathways leading to the production of this important organic acid in microorganisms.

## BIOSYNTHESIS PATHWAY

Kinoshita (1932) recognized that a filamentous fungus was able to produce itaconic acid and consequently described this species as *A. itaconicus*. The biosynthesis of itaconic acid was for a long time hotly debated, because it was not clear whether itaconic acid arises from a pathway including parts of the tricarboxylic acid (TCA) cycle or an alternative pathway via citramalate or the condensation of acetyl-CoA.

**FIGURE 1:**
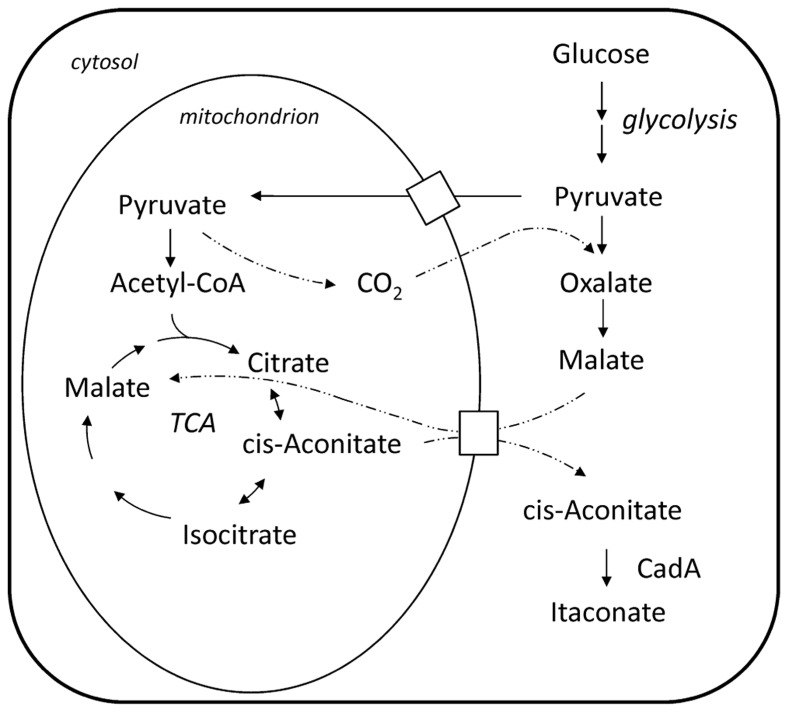
**Biosynthesis pathway of itaconate and its compartmentalization between cytosol and mitochondrion in the *A. terreus* cell; note: *cis*-aconitate transport is speculative**.

Bentley and Thiessen (1957a) proposed a pathway for the biosynthesis of itaconic acid, which is depicted in **Figure 1**. Starting from a sugar substrate like glucose the carbon molecules are processed via glycolysis to pyruvate. Then the pathway is split and part of the carbon is metabolized to Acetyl-CoA releasing a carbon dioxide molecule. The other part is converted to oxaloacetate so that the previously released carbon dioxide molecule is again incorporated. In the first steps of the citric acid cycle, citrate and *cis-*aconitate are formed. In the last step, the only itaconic acid pathway dedicated step, *cis-*aconitate decarboxylase (CadA) forms itaconic acid releasing carbon dioxide. This pathway was confirmed by tracer experiments with ^14^C and ^13^C labeled substrates (Bentley and Thiessen, 1957a; Winskill, 1983; Bonnarme et al., 1995) and also the necessary enzymatic activities have been all determined (Jaklitsch et al., 1991).

The formation of carboxylic acids, like citric and itaconic acid, involves the shuttling of intermediate metabolites between different intracellular compartments and utilizes the different enzymatic capabilities of the respective compartment. In case of itaconic acid the compartmentalization of the pathway was analyzed by fractionized cell extracts distinguishing the enzymatic activity of a mitochondrial from a cytosolic enzyme. It was found that the key enzyme of the pathway, CadA, is not located in the mitochondria but in the cytosol (Jaklitsch et al., 1991), whereas the enzymes preceding in the pathway, namely citrate synthase and aconitase, are found in the mitochondria. However, a residual level of aconitase and citrate synthase activity is also found in the cytosolic fraction. The proposed mechanism is that *cis-*aconitate is transported via the malate–citrate antiporter into the cytosol (Jaklitsch et al., 1991). However, so far it was not shown whether *cis-*aconitate makes use of the mitochondrial malate–citrate antiporter or uses another mitochondrial carrier protein to be translocated to the cytosol.

Besides *A. terreus*, itaconic acid is known to be produced also by other fungi like *U. zeae *(Haskins et al., 1955), *U. maydis *(Haskins et al., 1955; Klement et al., 2012), *Candida* sp. (Tabuchi et al., 1981), and *Rhodotorula* sp. (Kawamura et al., 1981). No further investigations exist about the underlying reaction principles leading to itaconic acid formation in those species. However, recent evidence (Strelko et al., 2011; Voll et al., 2012) points into the direction that CadA activity constitutes the general pathway toward the formation of itaconic acid in nature. Very recently, itaconic acid was detected in mammalian cells, where it was found in macrophage-derived cells (Strelko et al., 2011). Those cells also possess a CadA activity and have the ability to form itaconic acid *de novo*. But, up to now no specific gene encoding this enzymatic activity was identified in mammalian cells.

However, the physiological role of itaconic acid in mammalian cells is still unknown. Strelko et al. (2011) speculate on the role of itaconic acid as an inhibitor of metabolic pathways, because it is described as an enzymatic inhibitor. On the one hand, itaconic acid is known to inhibit isocitrate lyase (Williams et al., 1971; McFadden and Purohit, 1977), which is the crucial part of the glyoxylate shunt, and thus can act as an antibacterial agent. On the other hand, itaconic acid can inhibit fructose-6-phosphate 2-kinase (Sakai et al., 2004) and thus have a direct influence on the central carbon metabolism. In rats it was shown that a itaconate diet leads to a reduced visceral fat accumulation, because of a suppressed glycolytic flux (Sakai et al., 2004).

### ITACONIC ACID PATHWAY SPECIFIC ENZYMES AND GENES

**FIGURE 2:**
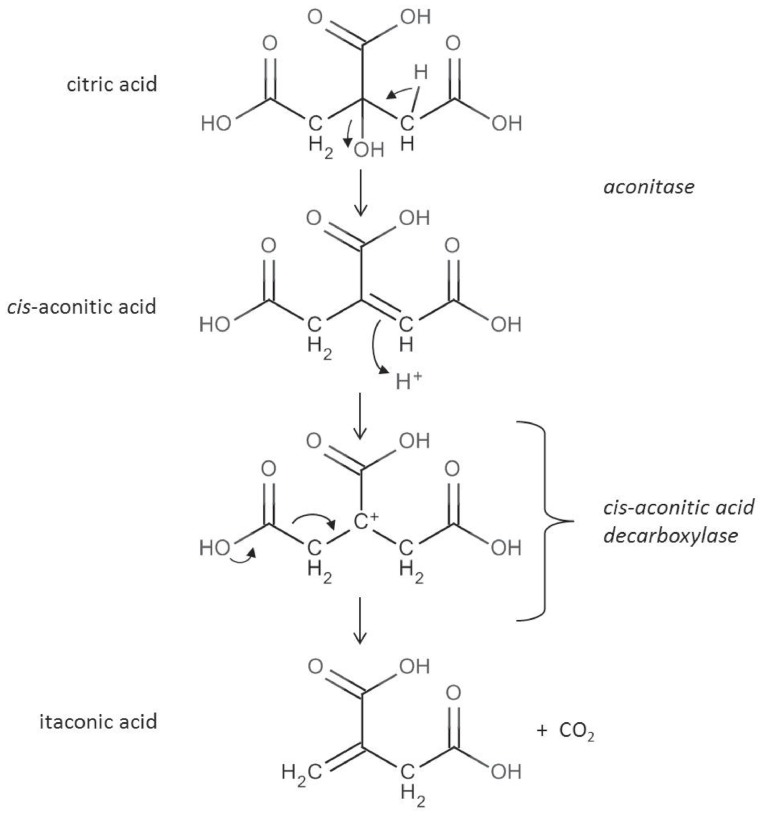
**In the citric acid cycle *cis-*aconitic acid is formed as an intermediate during the conversion of citric acid to isocitric acid. *cis-*aconitic acid is decarboxylated by the CadA enzyme to itaconic acid releasing CO_2_ (Bentley and Thiessen, 1957b)**.

The reaction catalyzed by the *cis-*aconitic acid decarboxylase was already described in 1957 (Bentley and Thiessen, 1957a,b). Subsequently performed ^13^C and ^14^C labeling experiments (Winskill, 1983; Bonnarme et al., 1995) confirmed the reaction scheme depicted in **Figure 2**. Itaconic acid is formed by an allylic rearrangement and decarboxylation from *cis-*aconitic acid removing either carbon C1 or C5 from the starting citric acid molecule (because of the symmetry of the molecule).

Furthermore, certain properties of the *A. terreus *CadA enzyme were determined: it has a *K*_m_ value of 2.45 mM (37°C, pH 6.2) and a pH optimum of 6.2 (Dwiarti et al., 2002). At pH 7.5 the activity drops significantly and is below 20% of the maximal value (Dwiarti et al., 2002). Until 2008, the sequence of the CadA protein was unknown, because the protein exhibits a general low stability. Kanamasa et al. (2008) were able to purify a substantial amount of the enzyme. By sequencing of the protein the N-terminal and four internal sequences were determined, which produced a single hit, ATEG_09971, in the genome database of *A. terreus*. The gene was named *cad1* and its protein product CadA. However, according to the nomenclature guidelines for *Aspergillus *it should rather be named *cadA* and CadA. The activity of the enzyme as a *cis-*aconitic acid decarboxylase (EC 4.1.1.6) was confirmed after heterologous expression of the gene in *Saccharomyces cerevisiae*. The CadA protein is a 490 amino acid protein (55 kDa) and has a high sequence identity with proteins from the MmgE/PrpD family, which includes 2-methylcitrate dehydratases. However, it is not clear, whether CadA has also a 2-methylcitrate dehydratase activity or whether a family member of the MmgE/PrpD class has also an activity as a *cis-*aconitic acid decarboxylase.

In contrast to the enzyme purification strategy, Li et al. (2011) used a transcriptomic approach to identify the *cadA *gene. A clone of the *A. terreus* strain NRRL1960 was cultivated at different conditions (pH, dissolved oxygen, etc.), which yielded different productivities and titers for itaconic acid. The conditions, which exhibited the highest difference in productivity and titer, were transcriptionally analyzed on a microarray with the assumption that genes involved in the itaconic acid pathway show an altered (higher) expression level during producing conditions. The *cadA* gene was highly scored in this analysis and thus can be identified in such an analysis. Interestingly another gene, encoding a mitochondrial carrier protein, was also highly scored in this analysis. This gene is located directly upstream of the *cadA* gene on the genome in *A. terreus*. Downstream of the *cadA* gene another transporter can be found which is annotated as a putative Major Facilitator Superfamily transporter. The mitochondrial carrier protein was detected in the transcriptomic analysis and was shown to have a direct positive influence on the itaconic acid production (Jore et al., 2011; van der Straat et al., 2012). However, the mechanism and substrates of this putative transporter are still unknown and its role needs to be clarified, but it can be speculated that intermediates of the biosynthesis pathway like *cis-*aconitic acid are transported with this protein.

The activity of the *cis-*aconitic acid decarboxylase is crucial for the performance of the whole itaconic acid biosynthesis pathway. In an itaconic acid overproducing strain, which was obtained by an selection on high itaconate levels (Yahiro et al., 1995), five times higher transcription levels of the *cadA* gene were found than in a comparable wild type strain but no change in the amino acid sequence was detected (Kanamasa et al., 2008). Expressing the *cadA* gene in *A. niger* under various constitutive promoters of different expression strength demonstrated that the itaconic acid productivity directly correlates with the *cadA* transcript level (Blumhoff et al., 2013). It can be concluded that a high transcriptional level of this gene is essential for an optimal production performance. A high transcriptional level of the gene might be necessary, because of a low stability of the enzyme *in vivo*, which was found to be rather unstable *in vitro *(Dwiarti et al., 2002; Kanamasa et al., 2008).

## CATABOLIZATION OF ITACONIC ACID

Much is known about the biosynthesis of itaconic acid and the underlying enzymatic mechanisms, but for a complete biochemical picture of a certain metabolite, also the knowledge about its degradation is necessary. Unfortunately, the information about the degradation pathway of itaconic acid is scarce. In mammalian cells (guinea pig and rat liver) it was found that itaconate is converted to itaconyl-CoA (Adler et al., 1957) and is further processed via citramalyl-CoA (Wang et al., 1961) to pyruvate and acetyl-CoA. Hereby, it was found that malonate has an inhibitory effect and an addition prevents the degradation of itaconic acid (Adler et al., 1957). The first step of this degradation pathway can be catalyzed by the ubiquitous succinyl-CoA synthetase (Adler et al., 1957; Nagai, 1963; Schürmann et al., 2011). The third step of the pathway is catalyzed by a citramalyl-CoA lyase, where genes from *Chloroflexus aurantiacus *(Friedmann et al., 2007) and *Pseudomonas putida *(Jain, 1996) have been cloned. However, no protein and gene sequence was identified so far, which can catalyze the second step of the degradation pathway, which is an itaconyl-CoA hydratase (Cooper and Kornberg, 1964).

## METABOLIC ENGINEERING OF THE ITACONIC ACID PATHWAY IN *A. terreus* AND *A. niger*

The levels of itaconic acid which were reached with *A. terreus* are currently limited to about 85 g/L. Although this is already a substantial amount it cannot be compared with the production of citric acid where titers over 200 g/L are steadily obtained in industrial processes. Transferred to the itaconic acid production a maximal theoretical titer of about 240 g/L should be achievable (Li et al., 2011). This goal could be reached by further breeding of currently existing strains or targeted genetic engineering.

In *A. terreus, *a gene was shown to influence the performance of itaconic acid production, which is a key enzyme of glycolysis. 6-phosphofructo-1-kinase is known to be inhibited by citrate and adenosine triphosphate (ATP). However, a truncated version of the *A. niger pfkA* gene was shown to exhibit a higher citric acid yield due to a reduced inhibition by citrate and ATP (Capuder et al., 2009). This truncated *pfkA* version had also a positive impact on the itaconic acid accumulation when expressed in *A. terreus* (Tevz et al., 2010). Another engineering approach deals with the intracellular oxygen supply. The production of itaconic acid requires continuous aeration and already a short interruption of oxygen decreases the itaconic acid yield. In order to reduce the sensitivity to oxygen a hemoglobin gene from *Vitreoscilla* was expressed in *A. terreus*. Indeed, the expression of this gene leads to an increased itaconic acid production. Furthermore, the strains exhibited a better recovery after the aeration was interrupted (Lin et al., 2004).

There is the possibility that the genetic make-up of *A. terreus *is not efficient enough to support the production of higher titers of organic acids. Therefore, a strategy is to genetically engineer the itaconic acid biosynthesis pathway into another host organism, which is already known to support the production of high titers of organic acids. As already mentioned, *A. niger* is such a candidate. The unique and crucial step in the biosynthesis pathway is the decarboxylation of *cis-*aconitic acid toward itaconic acid. When the *cadA* gene (Kanamasa et al., 2008) was characterized in *A. terreus* genetic engineering of the pathway into another organism became possible. Li et al. (2011) expressed the *A. terreus cadA *gene in *A. niger* strain AB 1.13. For this purpose, the *cadA *gene was placed under the control of the *A. niger gpdA *promoter, which enables a strong and constitutive expression. An *A. niger* strain which expresses the *cadA *gene alone has the ability to produce about 0.7 g/L itaconic acid. This level is not comparable with current production strains of *A. terreus*, but is a promising starting point for further engineering steps. Further attempts to rise the yield are to express genes like the above mentioned mitochondrial carrier protein together with the *cadA* gene (Jore et al., 2011; van der Straat et al., 2012).

## OUTLOOK

Itaconic acid as a renewable organic acid is of growing interest for the chemical industry, because of its potential to replace crude oil based products like acrylic acid. Up to now, the microorganism based processes were improved by classical strain breeding and optimizations of the fermentation strategies and conditions. Especially the knowledge about the biotechnological process including oxygen supply, media compositions, and different bioreactor systems was significantly expanded (Kuenz et al., 2012). Regarding the media composition, it was found that copper ions positively influence the itaconic acid production in a genetically engineered *A. niger *strain (Li et al., 2012). However, it is not understood which biochemical reactions are responsible or involved in such an effect. As already mentioned above, the biochemical reactions and effects of itaconic acid in the production hosts are not fully described. The catabolization pathway of itaconic acid requires further investigations in order to engineer a production host with a disabled degradation pathway. The effect of itaconic acid on other metabolic pathways is also of interest because the understanding of its physiological role can prevent undesired side effects (toxicity, health risk, pathway inhibition) and increase the safety of its use. Furthermore, it can be an interesting target for medical research because in mammalian cells it was detected in a metastatic tumor cell line (Strelko et al., 2011). Further knowledge about its role as an enzyme inhibitor can help to develop less-resistant enzyme varieties like in the case of the phosphofructokinase 2. Another target for further engineering is the CadA enzyme, which is described as an unstable protein. Prolonging its *in vivo* stability can help to increase the efficiency of existing production hosts. Also the genetic regulation of the itaconic acid pathway in *A. terreus* requires a profound analysis. Li et al. (2011) have shown that genes involved in the biosynthesis pathway (*cadA*) can be identified by transcriptomic approaches. However, nothing is known so far about the regulatory mechanisms leading to the expression of those genes.

The investigations on the molecular principles of itaconic acid synthesis revealed that *cis-*aconitic acid decarboxylase is the dedicated step in its biosynthesis in *A. terreus*. Genetic engineering of this enzymatic step also renders other microbial hosts like *A. niger* to producers of itaconic acid.

## Conflict of Interest Statement

The authors declare that the research was conducted in the absence of any commercial or financial relationships that could be construed as a potential conflict of interest.
